# Clinical significance of erythropoietin receptor expression in oral squamous cell carcinoma

**DOI:** 10.1186/1471-2407-12-194

**Published:** 2012-05-28

**Authors:** Yu-Tsai Lin, Hui-Ching Chuang, Chang-Han Chen, Gian Luca Armas, Han-Ku Chen, Fu-Min Fang, Chao-Cheng Huang, Chih-Yen Chien

**Affiliations:** 1 Department of Otolaryngology, Kaohsiung Chang Gung Memorial Hospital and Chang Gung University College of Medicine, 123 Ta-Pei Road, Niao-Song District, Kaohsiung, 833, Taiwan; 2 Kaohsiung Chang Gung Head and Neck Oncology Group, Cancer Center, Kaohsiung Chang Gung Memorial Hospital, Kaohsiung, Taiwan; 3 Center for Translational Research in Biomedical Sciences, Kaohsiung Chang Gung Memorial Hospital, Kaohsiung, Taiwan; 4 Operative unit of Otolaryngology, Hospital Santa Chiara, Trento, Italy; 5 Department of Pathology, Kaohsiung Chang Gung Memorial Hospital and Chang Gung University College of Medicine, Kaohsiung, Taiwan; 6 Department of Radiation Oncology, Kaohsiung Chang Gung Memorial Hospital and Chang Gung University College of Medicine, Kaohsiung, Taiwan

## Abstract

**Background:**

Hypoxic tumors are refractory to radiation and chemotherapy. High expression of biomarkers related to hypoxia in head and neck cancer is associated with a poorer prognosis. The present study aimed to evaluate the clinicopathological significance of erythropoietin receptor (EPOR) expression in oral squamous cell carcinoma (OSCC).

**Methods:**

The study included 256 patients who underwent primary surgical resection between October 1996 and August 2005 for treatment of OSCC without previous radiotherapy and/or chemotherapy. Clinicopathological information including gender, age, T classification, N classification, and TNM stage was obtained from clinical records and pathology reports. The mRNA and protein expression levels of EPOR in OSCC specimens were evaluated by Q-RT-PCR, Western blotting and immunohistochemistry assays.

**Results:**

We found that EPOR were overexpressed in OSCC tissues. The study included 17 women and 239 men with an average age of 50.9 years (range, 26–87 years). The mean follow-up period was 67 months (range, 2–171 months). High EPOR expression was significantly correlated with advanced T classification (*p* < 0.001), advanced TNM stage (*p* < 0.001), and positive N classification (*p* = 0.001). Furthermore, the univariate analysis revealed that patients with high tumor EPOR expression had a lower 5-year overall survival rate (*p* = 0.0011) and 5-year disease-specific survival rate (*p* = 0.0017) than patients who had low tumor levels of EPOR. However, the multivariate analysis using Cox’s regression model revealed that only the T and N classifications were independent prognostic factors for the 5-year overall survival and 5-year disease-specific survival rates.

**Conclusions:**

High EPOR expression in OSCC is associated with an aggressive tumor behavior and poorer prognosis in the univariate analysis among patients with OSCC. Thus, EPOR expression may serve as a treatment target for OSCC in the future.

## Background

Oral squamous cell carcinoma (OSCC), the sixth most common cancer worldwide, is the most frequently observed head and neck cancer in Southeast Asia [1], but despite improvements in treatment strategy, clinical outcomes are not satisfactory. The cancer recurs in a large proportion of patients, and the outcome of salvage therapies depends on the clinical stage of the disease when it recurs [2]. In Taiwan, many people in the lower socioeconomic bracket chew betel nut, which causes a significant threat to public health. In 2009, the rate of oral cancer was approximately 14.6 per 100,000 persons in Taiwan, and it is the fourth most common cancer in men (http://www.doh.gov.tw/statistic). In 2007, 22,560 new cases of oral cancer were reported in the United States, with approximately 5,370 deaths attributed to this disease [3].

In humans, erythropoietin receptors (EPORs) can be detected on the surface of erythroid progenitor cells in bone marrow [4,5] and in some non-hematopoietic cells such as vascular endothelial cells [6], retinal photoreceptors [7], liver stromal cells [8], neurons [9], and macrophages [10]. Also, recent studies have revealed that EPORs play an important role in biological activities such as physiological angiogenesis in wound healing and the female reproductive system, and provides protection against anoxic damage in the brain [11] and cardiovascular system [12,13].

Previous studies have reported that anemia is a poor prognostic factor for survival in patients with cancer [14] and that erythropoietin (EPO) treatment can improve the survival rate and quality of life of patients with anemia receiving chemoradiotherapy [15]. However, more recent studies have shown that EPO treatment may have different results in breast cancer and head and neck cancer [16]. Lai et al. [17] found that the level of EPO and EPOR can affect growth and metastasis of head and neck cancer. A biomarker that could predict the prognosis of oral cavity cancer would be a valuable addition to traditional imaging. The aim of the present study was to evaluate the clinicopathological significance of EPOR expression in patients with OSCC.

## Methods

### Patients and tissue samples

The study included 256 patients who underwent primary surgical resection between October 1996 and August 2005 for treatment of OSCC without previous radiotherapy and/or chemotherapy. Clinicopathological information including gender, age, tumor (T) classification, node (N) classification, and tumor, node, metastases (TNM) stage was obtained from clinical records and pathology reports. The TNM status was classified according to the 2002 American Joint Committee on Cancer Staging System. The treatment of oral cancer will be evitable to encounter the problems of local, regional or even distant failure. According to the guideline of our institute, the surgical salvage is the first choice if the tumor is operable. Otherwise, the patients will undergo salvage chemotherapy or chemoradiation if the tumor becomes unresectable although the prognosis of this group is dismal. To measure the expression of the EPOR in OSCC tissue and adjacent non-tumor tissues, fresh tissues were obtained from patients with OSCC biopsy specimens. These materials were histologically confirmed by frozen sections before quantitative RT-PCR, western blotting and IHC assays. The present study was approved by the Medical Ethics and the Human Clinical Trial Committee at Chang Gung Memorial Hospital, Taiwan.

### RNA extraction and quantitative RT-PCR (Q-RT-PCR)

Tissue samples were frozen in liquid nitrogen and stored at −80 °C prior to RNA extraction. The tissue was homogenized using a Mixer Mill Homogenizer (Qiagen, Crawley, West Sussex, UK). Total RNA was prepared from the frozen tissue samples using an RNeasy Mini Kit (Qiagen) according to the manufacturer’s instructions. The RNA (2 μg) was then reverse-transcribed into cDNA using SuperScript II Reverse Transcriptase (Invitrogen, Carlsbad, CA, USA). For Q-RT-PCR, *EPOR* Taq-Man probes (ABI) were used. Data were represented as mean ± s.d. To analyze the distribution of adjacent-non-tumor and tumor areas, we used the Wilcoxon signed rank test between two groups for statistical analysis. A *P*-value of less than 0.05 was considered statistically significant. *GAPDH* was used as an internal control for comparison and normalization of the data. Assays were performed in triplicate using an Applied Biosystems Model 7500-Fast instrument.

### Western blot analysis

Tissue protein extraction was carried out in frozen samples that were homogenized in radioimmunoprecipitation assay (RIPA) lysis buffer [50 mm Tris–HCl (pH 7.5), 150 mm NaCl, 1% NP-40, 0.5% Na-deoxycholate, and 0.1% sodium dodecyl sulfate (SDS)]. The protein concentration in each sample was estimated using a Bio-Rad Protein Assay (Bio-Rad, Hercules, CA, USA). Immunoblotting was performed according to standard procedures. The antibodies included polyclonal antibodies against EPOR and β-actin (monoclonal; Santa Cruz Biotechnology, Santa Cruz, CA, USA). The first antibodies were detected by incubation with secondary antibodies conjugated to HRP (Bio/Can Scientific, Mississauga, ON, Canada) and developed using Western Lighting chemiluminescence reagent (PerkinElmer Life Sciences Inc., Boston, MA, USA). X-ray films were used to detect the proteins.

### Immunohistochemistry

Adjacent noncancerous and OSCC tissue samples were selected by a pathologist based on diagnosis and microscopic morphology. The tissues were fixed in 10% buffered formalin, then embedded in paraffin and decalcified in 10% EDTA solution. Representative blocks of the formalin-fixed, paraffin-embedded tissues were cut into 4-mm sections and deparaffinized with xylene and rehydrated in a series of ethanol washes (100, 90, 80, and 70%). Slides were washed with phosphate-buffered saline (PBS) and treated with 3% H_2_O_2_ for 30 min to block endogenous peroxidase activity. Next, the sections were microwaved in 10 mM citrate buffer (pH 6.0) to unmask the epitopes. After antigen retrieval, the sections were incubated with diluted anti-EPOR antibody (sc-101444; monoclonal; 1:200; Santa Cruz Biotechnology) for 1 h followed by washing with PBS. Horseradish peroxidase/Fab polymer conjugate (PicTure-Plus kit; Zymed, South San Francisco, CA, USA) was then applied to the sections for 30 min followed by washing with PBS. Finally, the sections were incubated in diaminobenzidine for 5 min to develop the signals. A negative control that omitted the primary antibody was run simultaneously. Positive immunostaining was defined as cytoplasmic and/or membrane immunoreactivity. The level of reactivity in the immunostained tissues was evaluated independently by two pathologists who were blinded to the subjects’ clinical information, and scored into four groups according to the percentage and intensity of cytoplasmic and membrane staining. Specimens in which >30% of the cells were stained were scored as strongly positive (+++), specimens in which 10–30% of the cells were stained were scored as moderately positive (++), and those in which <10% of the cells were stained were scored as weakly positive (+). Specimens with no staining were scored as negative (−) [18]. A high level of EPOR expression was defined as staining of >10% of the tumor cells, and a low level of EPOR expression was defined as staining in <10% of the tumor cells.

### Statistical analysis

Several clinicopathological factors were evaluated, including gender, age (59 versus 60 years), T classification (T1, T2 versus T3, T4), N classification (negative versus positive), and TNM stage (stage I, II versus stage III, IV). Fisher’s exact test was used to evaluate the correlation between the clinicopathological variables and EPOR expression. A *p*-value less than 0.05 was deemed to be statistically significant. The clinicopathological variables and the expression of EPOR were taken into account for the analysis of survival based on the Kaplan-Meier method; statistical significance, defined as a *p* < 0.05, was assessed using the log-rank test. To determine the effect of particular prognostic factors on survival, a multivariate analysis was performed using Cox’s regression model.

## Results

### Immunohistochemical study of EPOR in OSCC tissues

The study included 239 men and 17 women with an average age of 50.9 years (range, 26–87 years). Of the 256 patients, 39 were classified as T1, 55 as T2, 64 as T3, and 98 as T4. The N classification was N0 in 153 patients (59.8%), N1 in 38 (14.8%), N2b in 48 (18.7%), N2c in 13 (5.1%), and N3 in 4 (1.6%) patients. The cancer stage was classified as I in 32 patients (12.5%), II in 34 (13.3%), III in 63 (24.6%), and as stage IV in 127 (49.6%) patients (Table 1). The mean follow-up period was 67 months (range, 2–171 months).

**Table 1:** Clinical profile and correlation between the clinicopathological features and EPOR expression in oral squamous cell carcinoma (OSCC)

**Variables**	**No. of patients**	**EPOR staining**		***p*-Value**
		Low expression	High expression	
Gender				
Male	239	133	106	0.128
Female	17	13	4	
Age, years				
<60	202	114	88	0.0758
≥60	54	32	22	
T classification				
T1, T2	94	73	21	<0.001*
T3, T4	162	73	89	
N classification				
Positive	103	46	57	<0.001*
Negative	153	100	53	
TNM stage				
I, II	66	60	6	<0.001*
III, IV	190	86	104	

EPOR, erythropoietin receptor; *statistically significant (*p* < 0.05).

**Figure 1:**
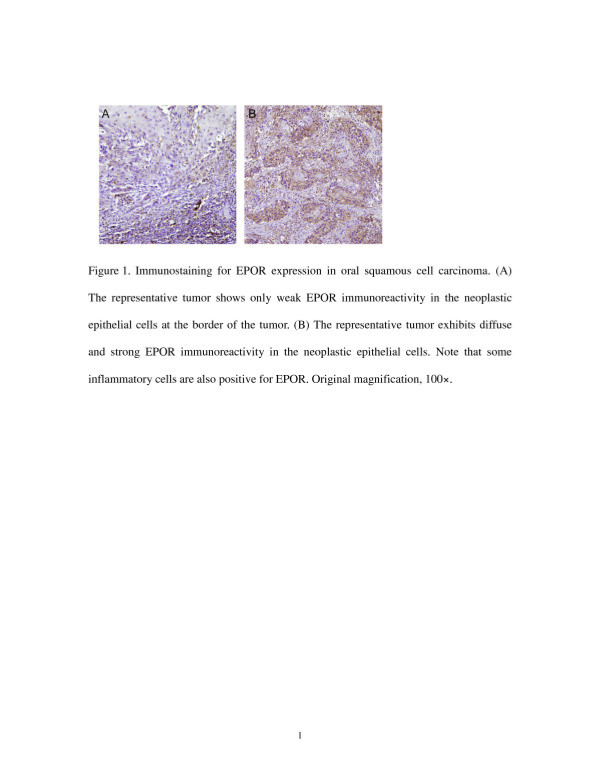
**Immunostaining for EPOR expression in oral squamous cell carcinoma.** (**A**) The representative tumor shows only weak EPOR immunoreactivity in the neoplastic epithelial cells at the border of the tumor. (**B**) The representative tumor exhibits diffuse and strong EPOR immunoreactivity in the neoplastic epithelial cells. Note that some inflammatory cells are also positive for EPOR. Original magnification, 100 × .

EPOR immunoreactivity was observed in the neoplastic epithelial cells of the OSCC (Figure 1A and B). Late-stage OSCC was characterized by diffuse and strong EPOR immunoreactivity in the neoplastic epithelial cells (Figure 1B). The immunostaining indicated low EPOR expression in 146 patients and high expression in 110. Table 1 shows EPOR expression in the cancer cells and the correlation with clinicopathological variables. High expression of EPOR was significantly correlated with the advanced T classifications (*p* < 0.001), positive N classification (*p* = 0.001), and an advanced TNM stage (*p* < 0.001).

### RT-PCR and western blot analyses

**Figure 2:**
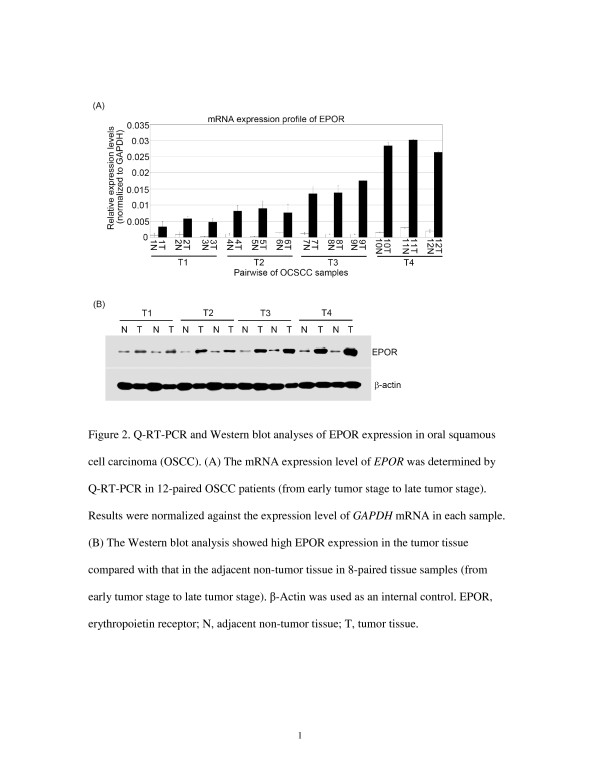
**Q-RT-PCR and Western blot analyses of EPOR expression in oral squamous cell carcinoma (OSCC).** (**A**) The mRNA expression level of *EPOR* was determined by Q-RT-PCR in 12-paired OSCC patients (from early tumor stage to late tumor stage). Results were normalized against the expression level of *GAPDH* mRNA in each sample. (**B**) The Western blot analysis showed high EPOR expression in the tumor tissue compared with that in the adjacent non-tumor tissue in 8-paired tissue samples (from early tumor stage to late tumor stage). β-Actin was used as an internal control. EPOR, erythropoietin receptor; N, adjacent non-tumor tissue; T, tumor tissue.

EPOR expression was measured in 12 paired samples of tumor tissue and adjacent non-tumor oral tissue (from early to late stage) using Q-RT-PCR. EPOR mRNA was overexpressed in all OSCC tissue samples, indicating a higher level of EPOR in the tumor tissue than in the adjacent non-tumor oral tissue after normalization using GAPDH (Figure 2A). To confirm this finding, EPOR protein expression was examined in 8 additional pairs of tumor and adjacent non-tumor tissue using the Western blotting technique. The results showed that EPOR expression was higher in the tumor tissue than in the adjacent non-tumor tissue in all four samples (Figure 2B), indicating that EPOR expression is low in adjacent non-tumor oral tissues and elevated in oral cancer tissues.

### Relationship between the 5-year survival rate and EPOR expression in OSCC

**Figure 3:**
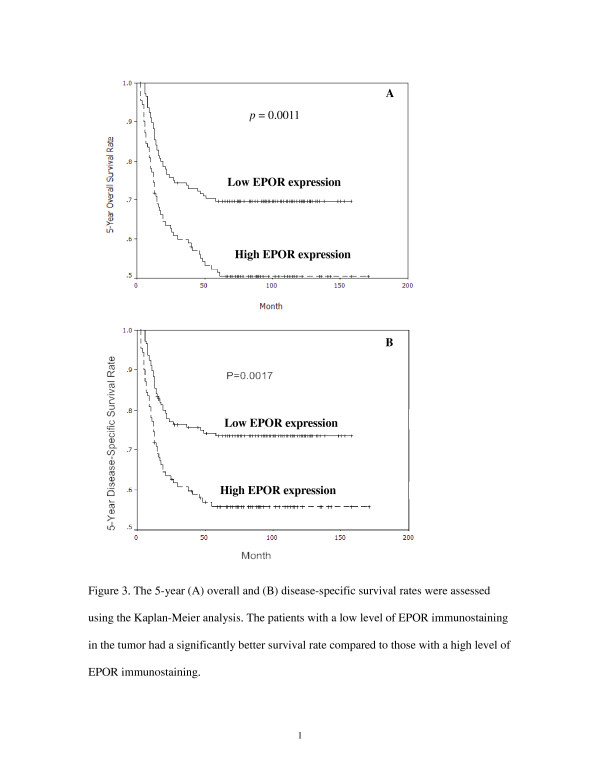
**The 5-year (a) overall and (b) disease-specific survival rates were assessed using the Kaplan-Meier analysis**. The patients with a low level of EPOR immunostaining in the tumor had a significantly better survival rate compared to those with a high level of EPOR immunostaining.

Patients with low levels of EPOR expression had a significantly higher 5-year overall survival rate than patients with high levels of EPOR (*p* = 0.0011; Figure 3A). Similarly, the 5-year disease-specific survival rate in patients with low EPOR expression was significantly higher than that in patients with high EPOR expression (*p* = 0.0017; Figure 3B). Furthermore, patients with advanced T classification, positive N classification, or advanced TNM stage had significantly lower 5-year overall and disease-specific survival rates compared to patients with early T classification, negative N classification, or early TNM stage (all *p* < 0.001; Tables 2 and 3). However, the multivariate analysis using Cox’s regression model revealed that only the T and N classifications were independent prognostic factors for the 5-year overall survival rate (*p* = 0.015) and 5-year disease-specific survival rate (*p* = 0.0025; Tables 4 and 5).

**Table 2:** Correlation between the 5-year overall survival rate and clinicopathological features in oral squamous cell carcinoma (OSCC)

**Variables**	**Number of patients**	**5-year overall survival rate (%)**	***p-*Value**
Gender			
Male	239	60.8	0.1
Female	17	81.9	
EPOR			
Low expression	146	69.6	0.0011*
High expression	110	51.5	
Age, years			
<60	202	64.5	0.0685
≥60	54	51.9	
T classification			
T1, T2	94	79.8	<0.001*
T3, T4	162	50.7	
N classification			
Negative	153	75	<0.001*
Positive	103	41.2	
TNM stage			
I, II	66	89.4	<0.001*
III, IV	190	52.2	

EPOR, erythropoietin receptor; *statistically significant (*p* < 0.05).

**Table 3:** Correlation between the 5-year disease-specific survival rate and clinicopathological features in oral squamous cell carcinoma (OSCC)

**Variables**	**Number of patients**	**5-Year disease-specific survival rate (%)**	***p-*Value**
Gender			
Male	239	64.8	0.174
Female	17	81.9	
EPOR			
Low expression	146	73.5	0.0017*
High expression	110	55.9	
Age, years			
<60	202	67.9	0.142
≥60	54	58.6	
T classification			
T1, T2	94	81.9	<0.001*
T3, T4	162	56.6	
N classification			
Negative	153	79.4	<0.001*
Positive	103	45.8	
TNM stage			
I, II	66	90.9	<0.001*
III, IV	190	57.2	

EPOR: erythropoietin receptor; *statistically significant (*p* < 0.05).

**Table 4:** Risk factors affecting 5-year overall survival rate determined by Cox’s regression analysis

**Variable**	**Relative risk**	**95% Confidence interval**	***p-*Value**
T classification			
T3, T4 vs. T1, T2	2.219	1.171–4.206	0.015
N classification			
Positive vs. Negative	2.651	1.679–4.187	<0.001

**Table 5:** Risk Factors affecting 5-year disease-specific survival rate determined by Cox’s regression analysis

**Variable**	**Relative risk**	**95% Confidence interval**	***p-*Value**
T classification			
T3, T4 vs. T1, T2	2.152	1.103–4.202	0.025
N classification			
Positive vs. Negative	2.651	1.766–4.763	<0.001

## Discussion

EPOR is highly expressed in the tumor cells of head and neck cancer, particularly in the hypoxic and infiltrating areas. EPOR rather than EPO may be upregulated by hypoxia in the tumor bed, and the high expression of EPOR in the tumor tissue may promote neck lymph node metastasis [19]. High EPOR expression in tumor tissue was found to be significantly correlated with high microvascular density in tongue cancer in an immunohistochemical study [18]. This finding suggests that the hypoxic microenvironment in a tumor may stimulate angiogenesis and promote tumor survival and growth.

EPOR in normal hematopoietic tissues is responsible for normal erythropoiesis. Abnormal EPOR function is associated with proliferative disorders of bone marrow such as primary erythrocytosis [15]. EPOR overexpression in tumor tissue is associated with a poor prognosis in a variety of malignancies, including breast cancer [20-23], melanoma [24], renal cell carcinoma [25], gastric cancer [26], pediatric tumors [27], and uterine and ovarian carcinoma [28]. However, only a few studies with limited cases have investigated the prognostic role of EPORs in OSCC. Recent studies have reported that EPO and EPORs are adverse prognostic factors for overall survival in patients with SCC of the tongue. Roh et al. [29] evaluated the prognostic value of hypoxia markers in 43 cases of T2-staged oral tongue cancer. They investigated the association between the 5-year survival rate and hypoxia biomarkers, such as HIF-1α, IF-2α, CA-9, GLUT-1, and EPOR, and found that EPOR was an independent biomarker in these patients, with a high propensity for regional metastasis Li et al. [18] reported that EPORs influenced the prognosis of carcinogenesis, angiogenesis, and the malignant progression in SCC of the tongue. Moreover, they found that EPORs were an independent prognostic marker. The results of our study are consistent with those of Li et al. [18] and suggest that EPOR is a significant prognostic indicator in patient with oral cancer. Thus, EPOR may play a role in tumor proliferation, apoptosis, and angiogenesis [30].

The TNM staging system is used to evaluate and predict the prognosis of patients with head and neck SCC (HNSCC). Clinicians develop therapeutic strategies according to the TNM stage of patients. Biomarkers that could reliably predict tumor behavior would be useful in the clinical setting. The clinical value of biomarkers related to angiogenesis and hypoxia in patients with HNSCC had been established in recent years [31-36]. These biomarkers were significantly correlated with tumor invasiveness and prognosis. The present study investigated EPOR expression in a large sample of patients with OSCC using immunohistochemical analysis. We found that a high EPOR expression was significantly correlated with advanced T classification, positive N classification, and advanced TNM stage. It represents the rapid proliferation of tumor cells may outpace the blood and nutrition supply. This probably leads to tumor necrosis and hypoxia in the microenvironment of tumor and may result into the higher level of EPOR. Furthermore, EPOR levels had a significant impact in the 5-year overall and 5-year disease-specific survival rates. We found that EPOR was overexpressed at both the mRNA and protein levels (data of Western blot assay) in OSCC tumors. Our findings are consistent with several reports showing that patients with oral cancer who had a high levels of EPOR expression had a poor 5-year overall survival rate [18,29]. To avoid the impact of other adjuvant treatment in the result of survival, the patients with high expression of EPOR still showed the poorer 5-year disease-specific survival significantly among patients underwent surgery only (*p* = 0.0079).

## Conclusions

High EPOR expression in patients with OSCC was associated with tumor progression in the present comprehensive study. These results suggest that EPOR expression is an important prognostic factor for OSCC. Thus, EPOR expression may serve as a treatment target for OSCC in the future.

## Competing interests

The authors declare that they have no competing interests.

## Authors' contributions

YTL, HCC, CHC, GLA, and CYC: collected the clinical data of patients, conceived the study design, carried out and coordinated immunohistochemical examinations of tumor specimens, and drafted the manuscript. HKC and CCH: participated in the interpretation of data and conducted immunohistochemistry analysis. CHC: performed the IHC staining and biochemical experiments. FMF: performed statistical data analysis. All authors read and approved the final manuscript.

## Pre-publication history

The pre-publication history for this paper can be accessed here:

http://www.biomedcentral.com/1471-2407/12/194/prepub

## References

[B1] Shah JP Singh B Keynote comment: why the lack of progress for oral cancer? Lancet Oncol 2006 7 356 357 10.1016/S1470-2045(06)70667-2 16648036

[B2] Chien CY Su CY Chuang HC Fang FM Huang HY Chen CM Chen CH Huang CC Angiopoietin-1 and −2 expression in recurrent squamous cell carcinoma of the oral cavity J Surg Oncol 2008 97 273 277 10.1002/jso.20930 18161864

[B3] Jemal A Siegel R Ward E Murray T Xu J Thun MJ Cancer statistics, 2007 CA Cancer J Clin 2007 57 43 66 10.3322/canjclin.57.1.43 17237035

[B4] D’Andrea AD Lodish HF Wong GG Expression cloning of the murine erythropoietin receptor Cell 1989 57 277 285 10.1016/0092-8674(89)90965-3 2539263

[B5] Jones SS D’Andrea AD Haines LL Wong GG Human erythropoietin receptor: cloning, expression, and biologic characterization Blood 1990 76 31 35 2163696

[B6] Anagnostou A Liu Z Steiner M Chin K Lee ES Kessimian N Noguchi CT Erythropoietin receptor mRNA expression in human endothelial cells Proc Natl Acad Sci USA 1994 91 3974 3978 10.1073/pnas.91.9.3974 8171022 PMC43705

[B7] Grimm C Wenzel A Groszer M Mayser H Seeliger M Samardzija M Bauer C Gassmann M Remé CE HIF-1-induced erythropoietin in the hypoxic retina protects against light-induced retinal degeneration Nat Med 2002 8 718 724 10.1038/nm723 12068288

[B8] Ohneda O Yanai N Obinata M Erythropoietin as a mitogen for fetal liver stromal cells which support erythropoiesis Exp Cell Res 1993 208 327 331 10.1006/excr.1993.1253 8359226

[B9] Nagai A Nakagawa E Choi HB Hatori K Kobayashi S Kim SU Erythropoietin and erythropoietin receptors in human CNS neurons, astrocytes, microglia, and oligodendrocytes grown in culture J Neuropathol Exp Neurol 2001 60 386 392 11305874 10.1093/jnen/60.4.386

[B10] Haroon ZA Amin K Jiang X Arcasoy MO A novel role for erythropoietin during fibrin-induced wound healing response Am J Pathol 2003 163 993 1000 10.1016/S0002-9440(10)63459-1 12937140 PMC1868246

[B11] Sakanaka M Wen TC Matsuda S Masuda S Morishita E Nagao M Sasaki R In vivo evidence that erythropoietin protects neurons from ischemic damage Proc Natl Acad Sci USA 1998 95 4635 4640 10.1073/pnas.95.8.4635 9539790 PMC22542

[B12] Wright GL Hanlon P Amin K Steenbergen C Murphy E Arcasoy MO Erythropoietin receptor expression in adult rat cardiomyocytes is associated with an acute cardioprotective effect for recombinant erythropoietin during ischemia-reperfusion injury FASEB J 2004 18 1031 1033 15059965 10.1096/fj.03-1289fje

[B13] Chong ZZ Kang JQ Maiese K Erythropoietin is a novel vascular protectant through activation of Akt1 and mitochondrial modulation of cysteine proteases Circulation 2002 106 2973 2979 10.1161/01.CIR.0000039103.58920.1F 12460881

[B14] Caro JJ Salas M Ward A Goss G Anemia as an independent prognostic factor for survival in patients with cancer: a systemic, quantitative review Cancer 2001 91 2214 2221 10.1002/1097-0142(20010615)91:12<2214::AID-CNCR1251>3.0.CO;2-P 11413508

[B15] Littlewood T Bajetta E Nortier JW Vercammen E Rapoport B Epoetin Alfa Study Group Effects of epoetin alfa on hematologic parameters and quality of life in cancer patients receiving nonplatinum chemotherapy: results of a randomized, double-blind, placebo-controlled trial J Clin Oncol 2001 19 2865 2874 11387359 10.1200/JCO.2001.19.11.2865

[B16] Bohlius J Langensiepen S Schwarzer G Seidenfeld J Piper M Bennett C Engert A Recombinant human erythropoietin and overall survival in cancer patients: results of a comprehensive meta-analysis J Natl Cancer Inst 2005 97 489 498 10.1093/jnci/dji087 15812074

[B17] Lai SY Childs EE Xi S Coppelli FM Gooding WE Wells A Ferris RL Grandis JR Erythropoietin-mediated activation of JAK-STAT signaling contributes to cellular invasion in head and neck squamous cell carcinoma Oncogene 2005 24 4442 4449 10.1038/sj.onc.1208635 15856028

[B18] Li HG Li JS Chen WL Wang L Wu DH Lin ZY Prognostic significance of erythropoietin and erythropoietin receptor in tongue squamous cell carcinoma Br J Oral Maxillofac Surg 2009 47 470 475 10.1016/j.bjoms.2009.06.001 19559511

[B19] Mohyeldin A Lu H Dalgard C Lai SY Cohen N Acs G Verma A Erythropoietin signaling promotes invasiveness of human head and neck squamous cell carcinoma Neoplasia 2005 7 537 543 10.1593/neo.04685 15967106 PMC1501166

[B20] Acs G Acs P Beckwith SM Pitts RL Clements E Wong K Verma A Erythropoietin and erythropoietin receptor expression in human cancer Cancer Res 2001 61 3561 3565 11325818

[B21] Acs G Zhang PJ Rebbeck TR Acs P Verma A Immunohistochemical expression of erythropoietin and erythropoietin receptor in breast carcinoma Cancer 2002 95 969 981 10.1002/cncr.10787 12209679

[B22] Arcasoy MO Amin K Karayal AF Chou SC Raleigh JA Varia MA Haroon ZA Functional significance of erythropoietin receptor expression in breast cancer Lab Invest 2002 82 911 918 12118093 10.1097/01.lab.0000020415.72863.40

[B23] Arcasoy MO Jiang X Haroon ZA Expression of erythropoietin receptor splice variants in human cancer Biochem Biophys Res Commun 2003 307 999 1007 10.1016/S0006-291X(03)01303-2 12878211

[B24] Selzer E Wacheck V Kodym R Schlagbauer-Wadl H Schlegel W Pehamberger H Jansen B Erythropoietin receptor expression in human melanoma cells Melanoma Res 2000 10 421 426 10.1097/00008390-200010000-00003 11095402

[B25] Westenfelder C Baranowski RL Erythropoietin stimulates proliferation of human renal carcinoma cells Kidney Int 2000 58 647 657 10.1046/j.1523-1755.2000.00211.x 10916088

[B26] Ribatti D Marzullo A Nico B Crivellato E Ria R Vacca A Erythropoietin as an angiogenic factor in gastric carcinoma Histopathology 2003 42 246 250 10.1046/j.1365-2559.2003.01581.x 12605644

[B27] Batra S Perelman N Luck LR Shimada H Malik P Pediatric tumor cells express erythropoietin and a functional erythropoietin receptor that promotes angiogenesis and tumor cell survival Lab Invest 2003 83 1477 1487 10.1097/01.LAB.0000090156.94795.48 14563949

[B28] Yasuda Y Musha T Tanaka H Fujita Y Fujita H Utsumi H Matsuo T Masuda S Nagao M Sasaki R Nakamura Y Inhibition of erythropoietin signalling destroys xenografts of ovarian and uterine cancers in nude mice Br J Cancer 2001 84 836 843 10.1054/bjoc.2000.1666 11259101 PMC2363820

[B29] Roh JL Cho KJ Kwon GY Ryu CH Chang HW Choi SH Nam SY Kim SY The prognostic value of hypoxia markers in T2-staged oral tongue cancer Oral Oncol 2009 45 63 68 10.1016/j.oraloncology.2008.03.017 18620902

[B30] Henke M Laszig R Rübe C Schäfer U Haase KD Schilcher B Mose S Beer KT Burger U Dougherty C Frommhold H Erythropoietin to treat head and neck cancer patients with anaemia undergoing radiotherapy: randomised, double-blind, placebo-controlled trial Lancet 2003 362 1255 1260 10.1016/S0140-6736(03)14567-9 14575968

[B31] Chien CY Su CY Hwang CF Chuang HC Hsiao YC Wu SL Huang CC The clinicopathological significance of CD105 expression in squamous cell carcinoma of hypopharynx Head Neck 2006 28 441 446 10.1002/hed.20364 16320363

[B32] Chien CY Su CY Chuang HC Fang FM Huang HY Chen CH Chen CM Huang CC Comprehensive study on the prognostic role of osteopontin expression in oral squamous cell carcinoma Oral Oncol 2009 45 798 802 10.1016/j.oraloncology.2008.12.006 19213594

[B33] Chuang HC Su CY Huang HY Chien CY Chen CM Huang CC High expression of CD105 as a prognostic predictor of early tongue cancer Laryngoscope 2006 116 1175 1179 10.1097/01.mlg.0000224338.56902.28 16826056

[B34] Marioni G Marino F Blandamura S D'Alessandro E Giacomelli L Guzzardo V Lionello M De Filippis C Staffieri A Neoangiogenesis in laryngeal carcinoma: angiogenin and CD105 expression is related to carcinoma recurrence rate and disease-free survival Histopathology 2010 57 535 543 10.1111/j.1365-2559.2010.03664.x 20955379

[B35] Chen CH Chien CY Huang CC Hwang CF Chuang HC Fang FM Huang HY Chen CM Liu HL Huang CY Expression of FLJ10540 is correlated with aggressiveness of oral cavity squamous cell carcinoma by stimulating cell migration and invasion through increased FOXM1 and MMP-2 activity Oncogene 2009 28 2723 37 10.1038/onc.2009.128 19525975

[B36] Chen CH Su CY Chien CY Huang CC Chuang HC Fang FM Huang HY Chen CM Chiou SJ Overexpression of beta2-microglobulin is associated with poor survival in patients with oral cavity squamous cell carcinoma and contributes to oral cancer cell migration and invasion Br J Cancer 2008 99 1453 61 10.1038/sj.bjc.6604698 18841160 PMC2579697

